# Genome Sequence of Grapevine Virus K, a Novel Vitivirus Infecting Grapevine

**DOI:** 10.1128/genomeA.00994-17

**Published:** 2017-09-14

**Authors:** Yeonhwa Jo, Myung-Kyu Song, Hoseong Choi, Jae-Seong Park, Jae-Wung Lee, Sen Lian, Bong Choon Lee, Won Kyong Cho

**Affiliations:** a Department of Agricultural Biotechnology, College of Agriculture and Life Sciences, Seoul National University, Seoul, Republic of Korea; b Grape Research Institute, Chungbuk Agricultural Research and Extension Services, Okcheon, Republic of Korea; c College of Crop Protection and Agronomy, Qingdao Agricultural University, Qingdao, Shandong, China; d Crop Foundation Division, National Institute of Crop Science, RDA, Wanju, Republic of Korea

## Abstract

Here, we report the genome sequence of grapevine virus K (GVK), a novel single-stranded RNA virus identified from a transcriptome of grapevine. The genome of GVK is 7,476 nucleotides in length and encodes 5 open reading frames. GVK is a putative member of the genus *Vitivirus* in the family *Betaflexiviridae*.

## GENOME ANNOUNCEMENT

The grapevine (*Vitis vinifera*) is a commercially important fruit crop worldwide that is used for wine production. Grapevines are clonally propagated by cutting and grafting. Thus, the infection rate of diverse viruses and viroids in grapevine is usually very high. To date, more than 70 different viruses infecting grape plants have been identified (1).

Recently, many novel viruses infecting grapevine have been identified with the help of next-generation sequencing techniques (2–4). Moreover, several recent studies have found that much plant transcriptome data contain viral sequences, which sometimes enables the assembly of the draft genome of a virus or viroid (5–7).

In the search for viruses and viroids from grapevine transcriptomes, we identified a novel virus from a transcriptome of the grapevine cultivar Lambrusco salamino (8). To summarize, three individual samples were pooled and used for total mRNA extraction. A cDNA library was prepared using the Illumina TruSeq RNA kit and single-end (85 bp) sequenced by the Illumina GAIIx platform. The raw data (3.3 Gb) for grapevine cultivar Lambrusco salamino deposited in the Sequence Read Archive (SRA) database (accession number ERR922631) were downloaded. The raw sequence reads were *de novo* assembled with Trinity version 2.0.6 with default parameters (9). The assembled contigs were analyzed with a BLAST search against the viral reference database (https://www.ncbi.nlm.nih.gov/genome/viruses).

We identified 143 contigs associated with viruses and viroids. Of these, three contigs were associated with a novel virus referred to as grapevine virus K (GVK) isolate Jo (GenBank accession number MF072319). GVK is a positive single-stranded RNA virus with a length of 7,476 nucleotides (nt). The obtained GVK genome sequence is a nearly full-length genome containing sequences for the 5′ and 3′ untranslated regions (80 and 77 nt in length, respectively). GVK encodes five open reading frames (ORFs), ORF1 (RNA-dependent RNA polymerase), ORF2 (unknown protein), ORF3 (movement protein), ORF4 (coat protein), and ORF5 (nucleic acid-binding protein). A BLASTp search using five GVK ORFs against NCBI’s nonredundant protein database revealed that ORF1 of GVK was homologous to the known grapevine virus A (GVA) with 52% protein sequence identity. No protein showed sequence similarity to ORF2 of GVK. ORF3 and ORF4 for GVK showed sequence similarity to GVA (50% protein identity) and grapevine virus D (GVD) (97% protein identity). The ORF5 of GVK showed sequence similarity to GVD (96% protein identity) with the *E* value (9e^−59^). Based on the BLASTp results, GVK could be a potential member of the genus *Vitivirus* in the family *Betaflexiviridae* along with GVA and GVD. Taken together, we identified and *de novo* assembled a novel virus referred to as GVK, a putative member of the genus *Vitivirus* from the grapevine transcriptome.

### Accession number(s).

The full-genome sequence of grapevine virus K isolate Jo has been submitted to GenBank under the accession number MF072319.
